# Splenectomy improves liver fibrosis via tumor necrosis factor superfamily 14 (LIGHT) through the JNK/TGF-β1 signaling pathway

**DOI:** 10.1038/s12276-021-00574-2

**Published:** 2021-03-03

**Authors:** Qing-shan Liang, Jian-Gang Xie, ChaoPing Yu, ZhuSheng Feng, JingChang Ma, Yuan Zhang, Dong Wang, JianGuo Lu, Ran Zhuang, Jikai Yin

**Affiliations:** 1 Department of General Surgery, The Second Affiliated Hospital of Air Force Military Medical University, 710038 Xi’an, Shaanxi China; 2 Department of Emergency, The First Affiliated Hospital of Air Force Military Medical University, 710032 Xi’an, Shaanxi China; 3 Department of Immunology, School of Basic Medical Sciences, Air Force Military Medical University, 710032 Xi’an, Shaanxi China; 4 Transplant Immunology Laboratory, School of Basic Medical Sciences, Air Force Military Medical University, 710032 Xi’an, Shaanxi China; 5 Institute of Medical Research, Northwest Polytechnic University, 710072 Xi’an, Shaanxi China

**Keywords:** Tumour-necrosis factors, Liver fibrosis, Hepatic stellate cells, Liver cirrhosis, Experimental models of disease

## Abstract

Splenectomy has been reported to improve liver fibrosis in patients with cirrhosis and hypersplenism. However, the mechanisms remain unclear. Tumor necrosis factor superfamily 14 (TNFSF14; also known as LIGHT) is highly expressed in the context of fibrosis and promotes disease progression in patients with fibrotic diseases such as pulmonary and skin fibrosis. Here, we determined whether splenectomy controls the production of LIGHT to improve liver fibrosis. Splenectomy reduced serum LIGHT levels in cirrhotic patients with hypersplenism and a ConA-induced liver fibrosis mouse model. Blocking LIGHT resulted in the downregulation of TGF-β1 in RAW264.7 cells. LIGHT treatment of RAW264.7 and JS1 cells in coculture regulated transforming growth factor-β1 (TGF-β1) expression through the activation of JNK signaling. Small interfering RNA-mediated silencing of lymphotoxin β receptor (LTβR) in macrophages resulted in pronounced decreases in the levels of fibrosis and αSMA in JS1 cells. These results indicated that LIGHT bound to LTβR and drove liver fibrosis in vitro. Blocking TGF-β1 abolished the effect of LIGHT in vitro. Furthermore, the administration of recombinant murine LIGHT protein-induced liver fibrosis with splenectomy, while blocking LIGHT without splenectomy improved liver fibrosis in vivo, revealing that the decrease in fibrosis following splenectomy was directly related to reduced levels of LIGHT. Thus, high levels of LIGHT derived from the spleen and hepatic macrophages activate JNK signaling and lead to increased TGF-β1 production in hepatic macrophages. Splenectomy attenuates liver fibrosis by decreasing the expression of LIGHT.

## Liver disease: Splenectomy reduces a factor that fuels fibrosis

Surgical removal of the spleen prevents release of a molecular signal that exacerbates liver fibrosis and ultimately contributes to the onset of cirrhosis. Several studies have indicated that splenectomy may protect liver function in cirrhosis patients, but the mechanism underlying this beneficial effect remains unclear. JiKai Yin of the Second Affiliated Hospital of Air Force Military Medical University, Shaanxi Xi’an, China, and colleagues have proposed a signaling protein called LIGHT as a likely factor. This protein is elevated in serum of patients with cirrhosis and mouse models of this disease. The authors subsequently identified molecular pathways triggered by LIGHT that fuel fibrosis. Treatments that interfere with LIGHT function prevent fibrosis, as does the sharp decrease in LIGHT levels that occurs following splenectomy. These results highlight a promising mechanism for protecting liver function in cirrhosis patients.

## Introduction

Clinical complications such as splenomegaly and hypersplenism often occur in patients with cirrhosis^1,2^. The observed association between the liver and spleen in terms of anatomy, histology, and immunity has been defined as the liver–spleen axis^3^. Previous studies have suggested that abnormal splenic functions occur through a variety of immunological mechanisms to promote liver fibrosis and the development of cirrhosis^4,5^. Aoyama et al.^6^ reported that spleen-derived lipocalin-2 has an important role in regulating immune tolerance in the liver during liver fibrosis development. Furthermore, both clinical and animal studies indicate splenic involvement in hepatic fibrosis mainly through the production and secretion of TGF-β1 by splenic macrophages^7,8^.

Splenectomy has been used to improve and treat fatal complications associated with portal hypertension in cirrhosis patients, such as bleeding and esophageal and gastric varices^3,9^. Additionally, there have been many reports showing that splenectomy may be an efficient method to improve liver functions and the prognosis of esophageal varices^10,11^. Recently, an animal study showed that splenectomy improved hepatic fibrosis by affecting the cytokines secreted by macrophages^12^. Nomura et al.^8^ reported that splenectomy reversed liver fibrosis and improved the systemic immune status in cirrhotic patients. However, the mechanism by which splenectomy improves hepatic fibrosis remains unclear.

As a member of the TNF receptor superfamily, LIGHT was first described as a protein secreted by activated T lymphocytes that was cleaved by matrix metalloprotein-9 (MMP-9)^13^. LIGHT exerts biological effects by binding to two receptors in the TNF receptor superfamily, herpesvirus entry mediator (HVEM, or TNFRSF14) and lymphotoxin β receptor (LTβR, or TNFRSF3), which are broadly expressed in macrophages, fibroblastic reticular cells, dendritic cells, neutrophils, and T and B cells^14,15^. LIGHT may drive fibrosis by increasing the number of macrophages at inflamed sites and increase macrophage expression of TGF-β, MMP-9, and IL-8^16,17^. Previous studies have shown that LIGHT promotes fibrosis in lung and skin tissues. Blocking the binding of LIGHT to either of its receptors improves the clinical symptoms of fibrosis in the lung and skin^18,19^. Additionally, Herro et al.^15^ found the role of LIGHT in maintaining or perpetuating fibrosis and considered LIGHT to be an attractive target for fibrosis therapies in patients with cirrhosis. However, the relationship between splenectomy and LIGHT in the improvement of liver fibrosis and the underlying mechanisms remain largely unclear.

In this study, we investigated the effects of splenectomy in both cirrhotic patients and a mouse model of ConA-induced liver fibrosis to determine whether LIGHT, JNK, and TGF-β1 were necessary for the protective effects of splenectomy against liver fibrosis. Additionally, the direct role of LIGHT in liver fibrosis was investigated by the administration of murine LIGHT protein in vivo.

## Materials and methods

### Patients and clinical specimens

Normal sera were obtained from 23 healthy volunteers. Serum was collected from 23 cirrhotic patients with hypersplenism 1 h before and 10 d after splenectomy at the Department of General Surgery in the Second Affiliated Hospital of Air Force Military Medical University (Xi’an, China). To ensure adequate power to detect a prespecified effect, the sample size was chosen using the Power and Sample Size Program. The levels of LIGHT, TGF-β1, IL-6, and TNF-α were measured by enzyme-linked immunosorbent assay (ELISA). Patients with primary liver cancer, congenital heart disease, diabetes, chronic respiratory diseases, alcohol abuse, or a history of drug use were excluded. There was no history of hepatocirrhosis or splenomegaly in any volunteer, and liver biochemical analyses were normal. All subjects provided informed consent, and the experimental procedures were approved by the Research Ethics Committee of the Second Affiliated Hospital of Air Force Military Medical University. Patient information is presented in Supplemental Material S1.

### Animal models of liver fibrosis with splenectomy

Six- to eight-week-old female C57BL/6 mice were purchased from the Laboratory Animal Center of Air Force Military Medical University. The mice were kept in a specific pathogen-free facility at 21 ± 2 °C with a 12-h dark/light cycle. Concanavalin A (ConA) was administered to the mice via the tail vein at a dose of 12.5 mg/kg weekly for 7 consecutive weeks. A total of 24 mice were randomly assigned to four groups: Blank, ConA model, ConA combined with sham, and ConA combined with splenectomy. Each group included six mice. The sham group was only subjected to an open-and-close abdominal operation. Splenectomy was conducted 1 day after the fifth ConA injection. To mimic immune damage in vivo, ConA injections were continued until the mice were sacrificed. All mice were sacrificed on the first day of the 8th week, and blood and liver tissue samples were collected. Alanine aminotransferase (ALT), aspartate aminotransferase (AST), and platelet (PLT) levels were analyzed by Servicebio Co. (Wuhan, China). Sera were collected from mice at various times during the course of the ConA injections (0, 2, 4, 5, and 7 weeks) and after splenectomy to measure the level of LIGHT. Liver tissues were subjected to immunofluorescence staining for F4/80 and TGF-β1, immunohistochemical staining for α-smooth muscle actin (αSMA), and Sirius red staining. The experimental procedures and animal care were carried out strictly in accordance with the related ethical regulations of Air Force Military Medical University. There were no animals excluded from analyses, and no blinding was carried out, but the data analysts were blinded to the groupings.

### Splenectomy

All mice were subjected to isoflurane anesthesia before splenectomy. Splenectomy was performed as described previously^20^. All mice were euthanized on the first day of the 8th week, and surgical procedures were conducted under completely sterile conditions.

### Cell lines and transwell coculture assays

The murine hepatic macrophage cell line RAW264.7 and murine hepatic stellate cell line JS1 were obtained from the Department of Immunology of Air Force Military Medical University. All cells were cultured in DMEM with 10% FBS. The cell lines were verified to be authentic.

Recombinant murine LIGHT protein (rLIGHT) was used to stimulate RAW264.7 cells for 0, 6, 12, and 24 h to determine the optimal time for TGF-β1 production. TGF-β 1 mRNA was measured by qPCR. The expression of JNK and P-JNK in RAW264.7 cells was measured by western blotting.

In addition, LTβR-Ig (Fc-tagged recombinant mouse LTβR protein, LTβR-Ig) was used to block LIGHT in the coculture assay. Splenocytes from C57BL/6 mice were seeded in the upper chambers (5 × 10^5^) of a six-well Transwell plate, while 1 × 10^6^ RAW264.7 cells were seeded in the lower chambers. ConA (1.5 µg/ml; Sigma Chemical Co.) was added to activate T cells, and 10 μg/ml LTβR-Ig and control IgG were added to the upper chambers and incubated for 12 h^21^. The expression of TGF-β1 in RAW264.7 cells was measured by qPCR and western blotting.

For LIGHT stimulation, rLIGHT-treated RAW264.7 cells (5 × 10^5^) were seeded in the upper chambers of a six-well Transwell plate (Corning, Costar 3422), while 1 × 10^6^ JS1 cells were plated in the lower chambers. SP600125 (P-JNK inhibitor) was added to RAW264.7 cells 15 min before coculture to assess the regulatory effect of JNK signaling on TGF-β1 and αSMA expression. Cells were incubated for 12 h to examine whether TGF-β1 produced by rLIGHT-stimulated RAW264.7 cells affected the functions of JS1 cells. Additionally, the levels of TGF-β1 were measured in the supernatant of JS1 cells from the coculture assay. rLIGHT (Cat. No. 1794-LT/CF, R&D Systems) was used at a concentration of 100 ng/ml, and SP600125 (Cat. No.: HY-12041, MedChemExpress) was used at a concentration of 10 µM.

### In vitro knockdown of LTβR and HVEM

For the in vitro knockdown of LTβR and HVEM in RAW264.7 cells, siRNAs targeting murine LTβR and HVEM and control nonsense siRNA were transfected into RAW264.7 cells using Lipofectamine 3000 (Thermo Scientific, Waltham, MA). After LTβR and HVEM knockdown in RAW264.7 cells, the Transwell coculture assay was performed as described above. The level of TGF-β1 in RAW264.7 cells was measured by western blotting and immunofluorescence staining. The level of TGF-β1 in the culture medium of JS1 cells was measured by ELISA (Baizhoubio, Qingdao, China). αSMA expression in JS1 cells was measured by western blotting. All siRNAs were purchased from Guangzhou RiboBio Co., Ltd. (Guangzhou, China). siRNA vectors were added to the culture medium at a concentration of 100 nM. The siRNA targeting sequences were as follows: control nonsense siRNA: CCCTCGAATGTGAATGGAA; siHVEM: GCAAATGGCCTGAGCAAGT; and siLTβR: GGACACTTCCAGAACACTT.

### In vitro TGF-β1 inhibition

The coculture assay included three groups: the control group, which contained RAW264.7 and JS1 cells; the rLIGHT group, in which RAW264.7 and JS1 cells were stimulated by rLIGHT; and the TGF-β1-NAB (anti-TGF-β1-neutralizing antibody) group, in which RAW264.7 and JS1 cells were treated with rLIGHT and the anti-TGF-β1-neutralizing antibody. RAW264.7 cells were pretreated with rLIGHT and/or anti-TGF-β1-neutralizing antibody for 12 h and then cocultured with JS1 cells in the Transwell system. The levels of αSMA in JS1 cells were measured by western blotting and immunofluorescence staining. The concentration of both the anti-TGF-β1-neutralizing antibody (Cat. No.: MAB1835-SP, R&D Systems) and rLIGHT protein (Cat. No.: 1794-LT/CF, R&D Systems) was 100 ng/ml.

### Effects of recombinant murine LIGHT on liver fibrosis in vivo

A total of 24 C57BL/6 mice were randomly assigned to four groups. Except for mice in the control group, all mice received a weekly dose of 12.5 mg/kg body weight ConA via tail vein injection. Splenectomy was conducted 1 day after the fifth ConA injection. Beginning in the 6th week, the rLIGHT group received a weekly dose of 10 µg of rLIGHT, while the ConA group received ConA. In addition, LIGHT was blocked by the administration (i.p.) of 100 µg of recombinant LTβR weekly for 7 consecutive weeks^16^.

The mice were sacrificed on the first day of the 8th week, and liver tissue samples were subjected to immunofluorescence staining for F4/80+ and TGF-β1+ macrophages, immunohistochemical staining for αSMA, and Sirius red staining. Western blotting was used to measure the expression of TGF-β1 and αSMA in liver tissue samples.

### Histological staining

Sirius red staining, immunohistochemical staining, and immunofluorescence staining were performed as described previously^22,23^. Sirius red staining solution was purchased from Servicebio (Wuhan, China). Antibodies against murine F4/80 and TGF-β1 were used to immunofluorescently label macrophages and TGF-β1+ macrophages in the liver tissue of mice. Antibodies against murine TGF-β1 and αSMA were used to immunofluorescently label TGF-β1 in RAW264.7 cells and αSMA in JS1 cells, respectively. The following primary antibodies were used: F4/80 (gb11027; Servicebio, Wuhan, China), TGF-β1 (ab106582, Abcam, Cambridge, UK), and αSMA (sc-53142; Santa Cruz Biotechnology).

### Quantitative reverse transcription PCR (qPCR)

qPCR analysis was performed as described previously^23^. The data were analyzed by SDS 2.1 software. For all target genes, the relative abundance was determined by the comparative cycle threshold Ct method and normalized to GAPDH mRNA levels. The primers are presented in Supplemental Material S2.

### Western blot analysis

Whole-cell lysates of liver tissue or cells were obtained using RIPA lysis buffer containing protease and phosphatase inhibitors. Protein samples were separated by 5% or 10% SDS-PAGE. Then, the proteins were transferred onto a polyvinylidene fluoride membrane and incubated with primary antibodies at 4 °C overnight. Corresponding secondary antibodies were applied to the membrane and incubated for 1 h. Protein bands were visualized by chemiluminescence. The following primary antibodies were used: JNK (sc-571; Santa Cruz Biotechnology), P-JNK (sc-6254; Santa Cruz Biotechnology), TGF-β1 (ab106582, Abcam), αSMA (sc-53142; Santa Cruz Biotechnology), and β-actin (sc-47778, Santa Cruz Biotechnology).

### Statistical analysis

The data are expressed as the means ± standard error of the mean (SEM). For comparison of two groups, Student’s *t*-test was used. One-way ANOVA followed by the Tukey multiple comparison test was used to compare the differences between three or more groups. A value of *P* < 0.05 was considered statistically significant. Statistical analyses were performed using GraphPad Prism 5 (GraphPad Software Inc., San Diego, CA).

## Results

### Splenectomy reduces the serum level of LIGHT in cirrhotic patients

**Fig. 1:**
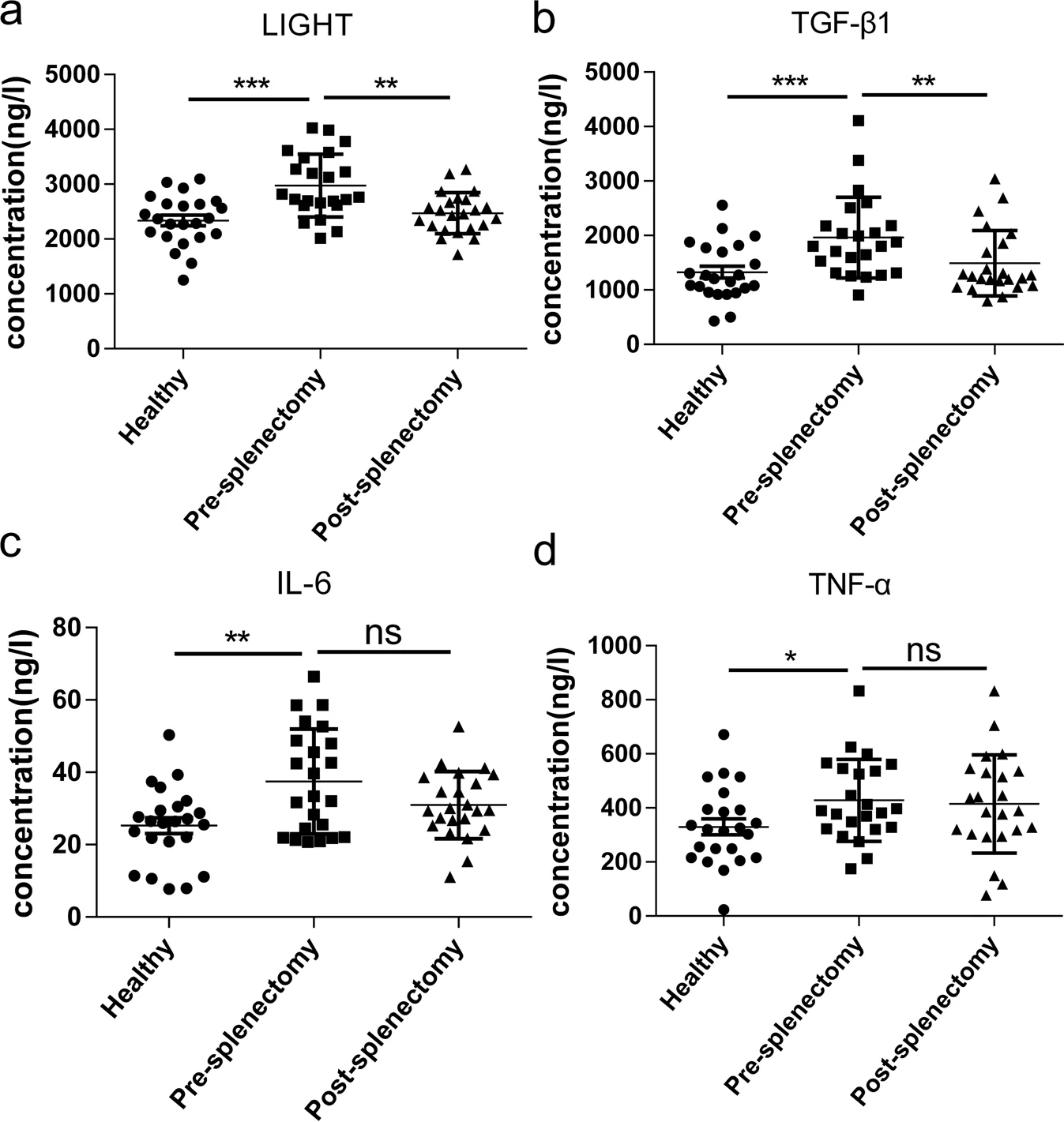
Effect of splenectomy on the profibrotic factors LIGHT, TGF-β1, IL-6, and TNF-α. **a–d** Serum concentrations of LIGHT, TGF-β1, IL-6, and TNF-α in healthy controls. Healthy (healthy volunteers), presplenectomy patients (cirrhotic patients before splenectomy), and postsplenectomy patients (cirrhotic patients after splenectomy). Cohort 3: *n* = 23, means ± SEM, *P < 0.05, **P < 0.01, ***P < 0.001.

ELISA was used to analyze the serum levels of LIGHT and other fibrotic markers, including TGF-β1, IL-6, and TNF-α, in healthy volunteers and cirrhotic patients before and after splenectomy. The serum levels of LIGHT, TGF-β1, IL-6, and TNF-α were all significantly increased in presplenectomy patients compared to healthy individuals (Fig. 1a–d), and LIGHT was more significantly increased in presplenectomy patients than in the other patients (*P* < 0.001).

However, compared with those of presplenectomy patients, LIGHT and TGF-β1 levels were decreased significantly in patients after splenectomy (Fig. 1a, b), while no significant difference in IL-6 or TNF-α was observed after splenectomy (*P* > 0.05) (Fig. 1c, d).

### The ConA-induced liver fibrosis and thrombocytopenia animal model is suitable for analyzing the effect of splenectomy on liver fibrosis and hypersplenism

**Fig. 2:**
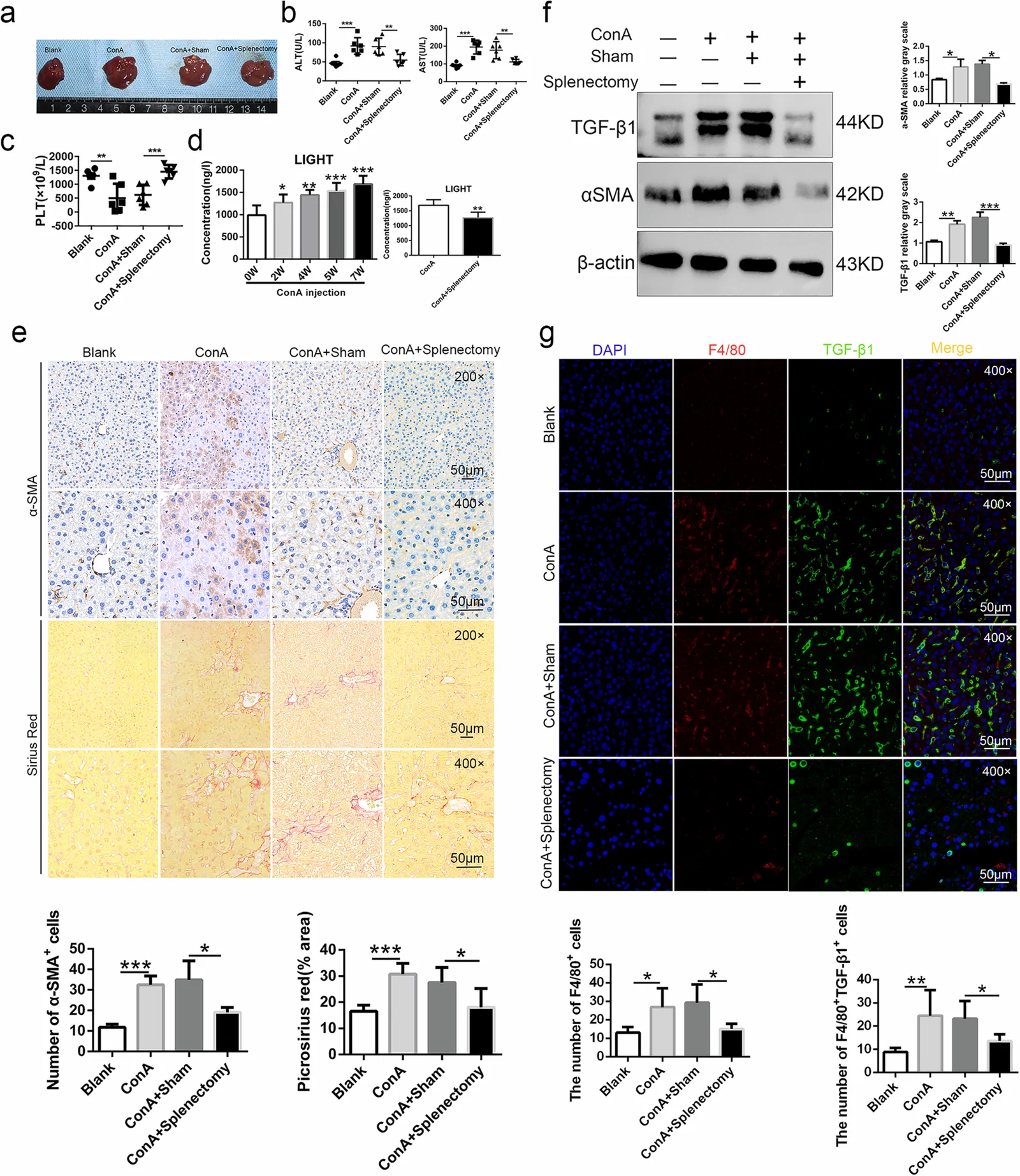
Morphological features, histological analysis of liver tissues, and serum levels of alanine aminotransferase (ALT), aspartate aminotransferase (AST), platelet (PLT), and LIGHT levels in mice in the four treatment groups. **a** Gross images of liver tissues. **b**–**d** Serum levels ALT, AST, PLT, and LIGHT. **e** Immunohistochemical analysis of αSMA expression and Sirius red staining of liver tissues in the Blank, ConA, sham, and splenectomy groups. Magnification: ×200 and ×400. **f** Levels of TGF-β1 and αSMA in liver tissues in the Blank, ConA, ConA+sham, and ConA+splenectomy groups. **g** Immunofluorescence staining of F4/80+ and F4/80+/TGF-β1+ macrophages in liver tissues in the Blank, ConA, sham, and splenectomy groups. Magnification: ×400. Blank (Normal mice), ConA (ConA-treated mice), ConA + Sham (ConA + Sham-treated mice), and ConA + Splenectomy (ConA + splenectomy-treated mice). ConA, concanavalin A; in the animal studies, *n* = 6/group. In all panels, scale bars = 50 µm. The relative grayscale value of the protein band was measured with ImageJ software and normalized to β-actin. The data (means ± SEM) were obtained from triplicate experiments (unpaired two-sample Student’s *t*-test and one-way ANOVA with Tukey’s multiple comparison test, *P < 0.05, **P < 0.01, ***P < 0.01).

After treatment of the mice with ConA for 7 continuous weeks, the liver surface was gray and granular in the ConA and sham groups, whereas mice subjected to splenectomy had smooth hepatic surfaces (Fig. 2a). ALT and AST were significantly increased (*P* < 0.001) following ConA treatment but markedly declined after splenectomy (*P* < 0.01) (Fig. 2b). The platelet count was markedly decreased after ConA treatment (*P* < 0.01) (Fig. 2c), which was concomitant with significantly increased serum ALT and AST levels, resembling hypersplenism with cirrhosis in humans. Most importantly, weekly injection of ConA obviously increased the serum concentration of LIGHT, which then decreased significantly after splenectomy (Fig. 2d). Moreover, immunohistochemical staining of αSMA revealed a significant increase in the number of activated myofibroblasts after ConA treatment (*P* < 0.001), while splenectomy significantly decreased the number of these cells (*P* < 0.05), and the Sirius red staining results were in accordance with the immunohistochemical staining results (Fig. 2e). The protein levels of TGF-β1 and αSMA were significantly increased after treatment with ConA, but splenectomy markedly decreased these levels (Fig. 2f). Additionally, immunofluorescence staining revealed increases in the percentages of F4/80+ and F4/80+/TGF-β1+ macrophages after ConA treatment, which were reduced by splenectomy (*P* < 0.05) (Fig. 2g).

### LIGHT induces JNK phosphorylation and increases TGF-β1 in macrophages

**Fig. 3:**
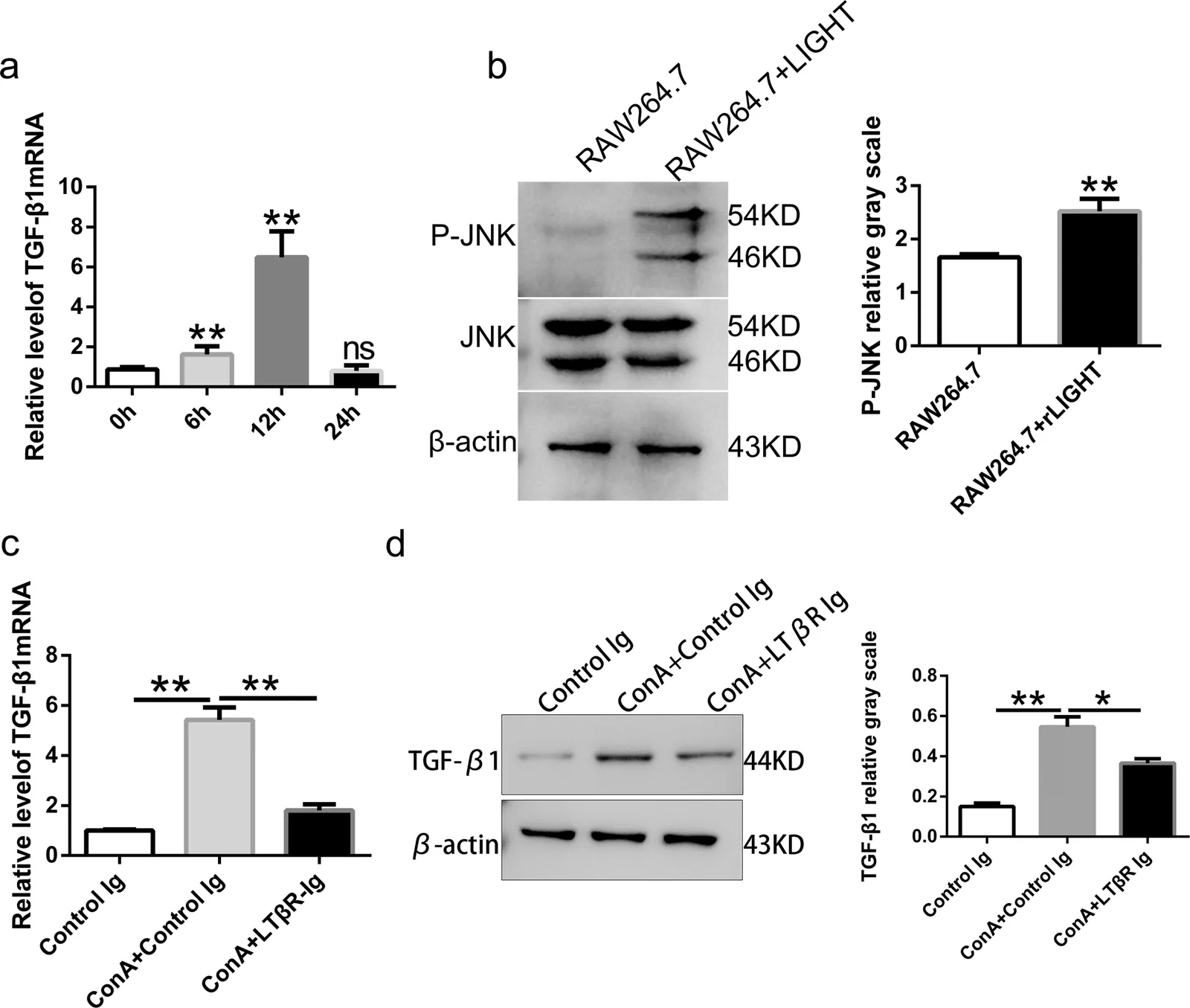
The levels of TGF-β1, P-JNK, and JNK in RAW264.7 cells. **a** mRNA level of TGF-β1 in RAW264.7 cells after treatment with rLIGHT for various times. **b** Protein levels of P-JNK and JNK in RAW264.7 cells after treatment with rLIGHT. **c** mRNA level of TGF-β1 in RAW264.7 cells after blocking LIGHT in splenocytes. **d** Protein level of TGF-β1 in RAW264.7 cells after blocking LIGHT in splenocytes. mRNA expression was examined by qPCR and normalized to GAPDH expression. The relative grayscale value of the protein band was measured with ImageJ software and normalized to β-actin. All data (means ± SEM) were obtained from triplicate experiments (one-way ANOVA with Tukey’s multiple comparison test, *P < 0.05, **P < 0.01).

To confirm whether LIGHT regulated liver fibrosis, the murine haptic macrophage cell line RAW264.7 was used for in vitro experiments. Because macrophages have a major role in promoting liver fibrosis^24^ and LIGHT was significantly increased in both cirrhotic patients and liver fibrosis animal models, we hypothesized that macrophages may be affected by LIGHT. First, we stimulated RAW264.7 cells with rLIGHT for 0, 6, 12, and 24 h to determine the optimal stimulation time. TGF-β1 mRNA levels were significantly upregulated at 12 h (*P* < 0.01) (Fig. 3a). Because TGF-β1 production correlates with P-JNK activity^25^, we postulated that TGF-β1 expression may be regulated by alterations in JNK signaling after rLIGHT treatment. Surprisingly, the level of phosphorylated JNK (P-JNK) was also significantly upregulated in the cells (*P* < 0.01) (Fig. 3b). Conversely, blocking LIGHT with LTβR-Ig in splenocyte and RAW264.7 cell cocultures resulted in decreased levels of TGF-β1 in RAW264.7 cells (Fig. 3c, d).

### LIGHT upregulates TGF-β1 in vitro to promote liver fibrosis through the JNK/P-JNK signaling pathway

**Fig. 4:**
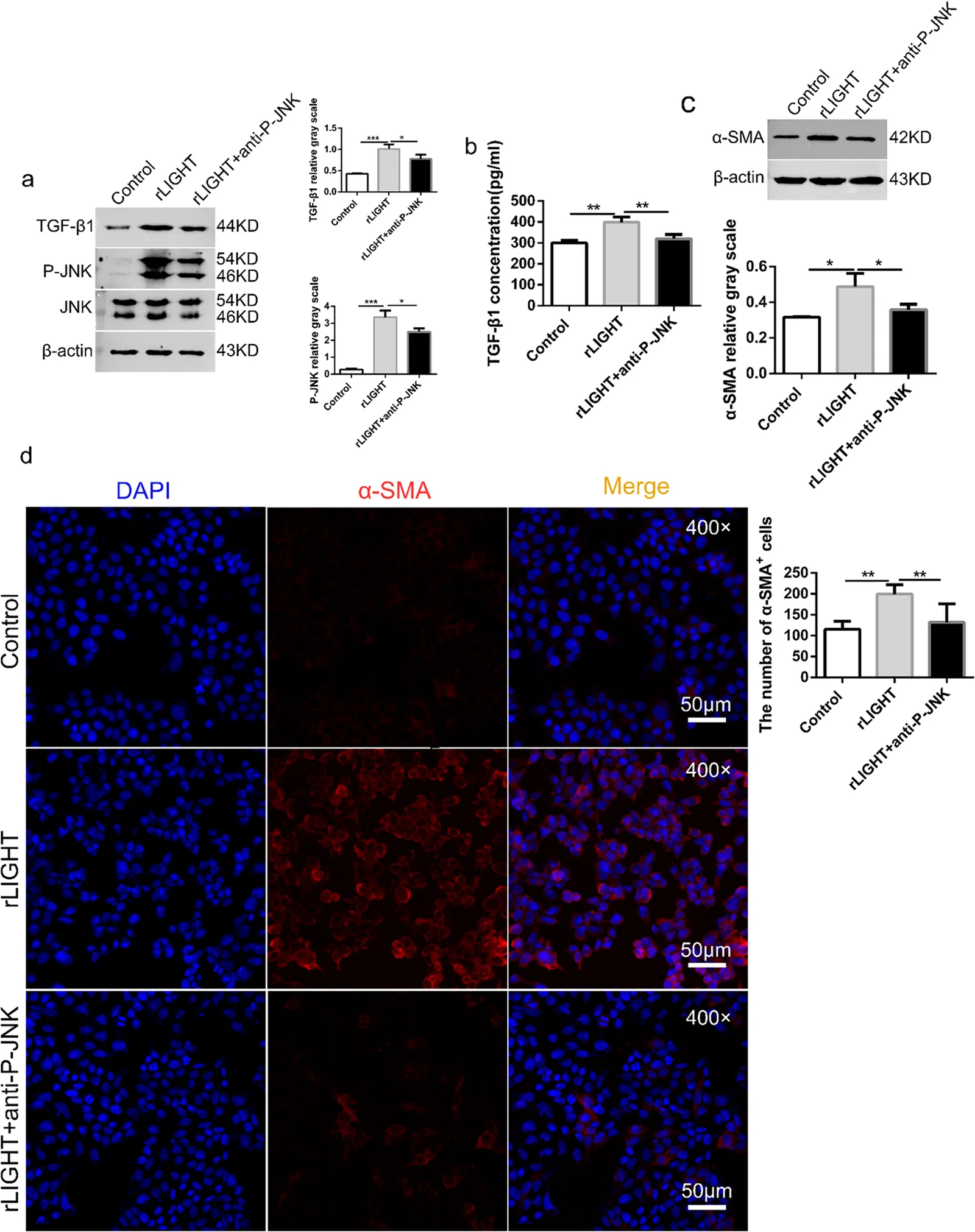
rLIGHT upregulates TGF-β1 expression in vitro to promote liver fibrosis through the JNK signaling pathway. **a** The levels of TGF-β1, P-JNK, and JNK in RAW264.7 cells. **b** The levels of TGF-β1 in the culture medium of JS1 cells. **c** The levels of αSMA in JS1 cells. **d** Immunofluorescence staining of αSMA and quantitative analysis of αSMA-positive cells among JS1 cells. Magnification: ×400. Control (RAW264.7 cells), rLIGHT (RAW264.7 cells + rLIGHT), rLIGHT + anti-P-JNK (RAW264.7 cells + rLIGHT + anti-P-JNK). Scale bar, 50 µm. mRNA expression was examined by qPCR and normalized to GAPDH expression. The relative grayscale value of the protein band was measured with ImageJ software and normalized to β-actin. The data (means ± SEM) were obtained from triplicate experiments (one-way ANOVA with Tukey’s multiple comparison test, *P< 0.05, **P < 0.01, and ***P < 0.001).

To explore the potential chemotactic effect of LIGHT in liver fibrosis, we used a Transwell coculture assay with RAW264.7 cells in the upper chamber and JS1 cells in the lower chamber. The expression of TGF-β1 and P-JNK in RAW264.7 cells was upregulated by LIGHT stimulation (*P* < 0.001) (Fig. 4a). Because TGF-β1 passed through the pores of the Transwell inserts from the upper to lower chambers, we hypothesized that the level of TGF-β1 was increased in JS1 cell culture medium. ELISA analysis showed that TGF-β1 was significantly increased in JS1 cell culture medium (*P* < 0.01) (Fig. 4b). Additionally, western blotting showed that the expression of αSMA was significantly upregulated in JS1 cells (*P* < 0.05) (Fig. 4c). Immunofluorescence analysis also showed a significant increase in the number of αSMA-positive cells among JS1 cells (*P* < 0.01) (Fig. 4d). In contrast, the addition of the JNK inhibitor SP600125 to RAW264.7 cells reversed these effects.

### LIGHT uses LTβR, but not HVEM to drive liver fibrosis

**Fig. 5:**
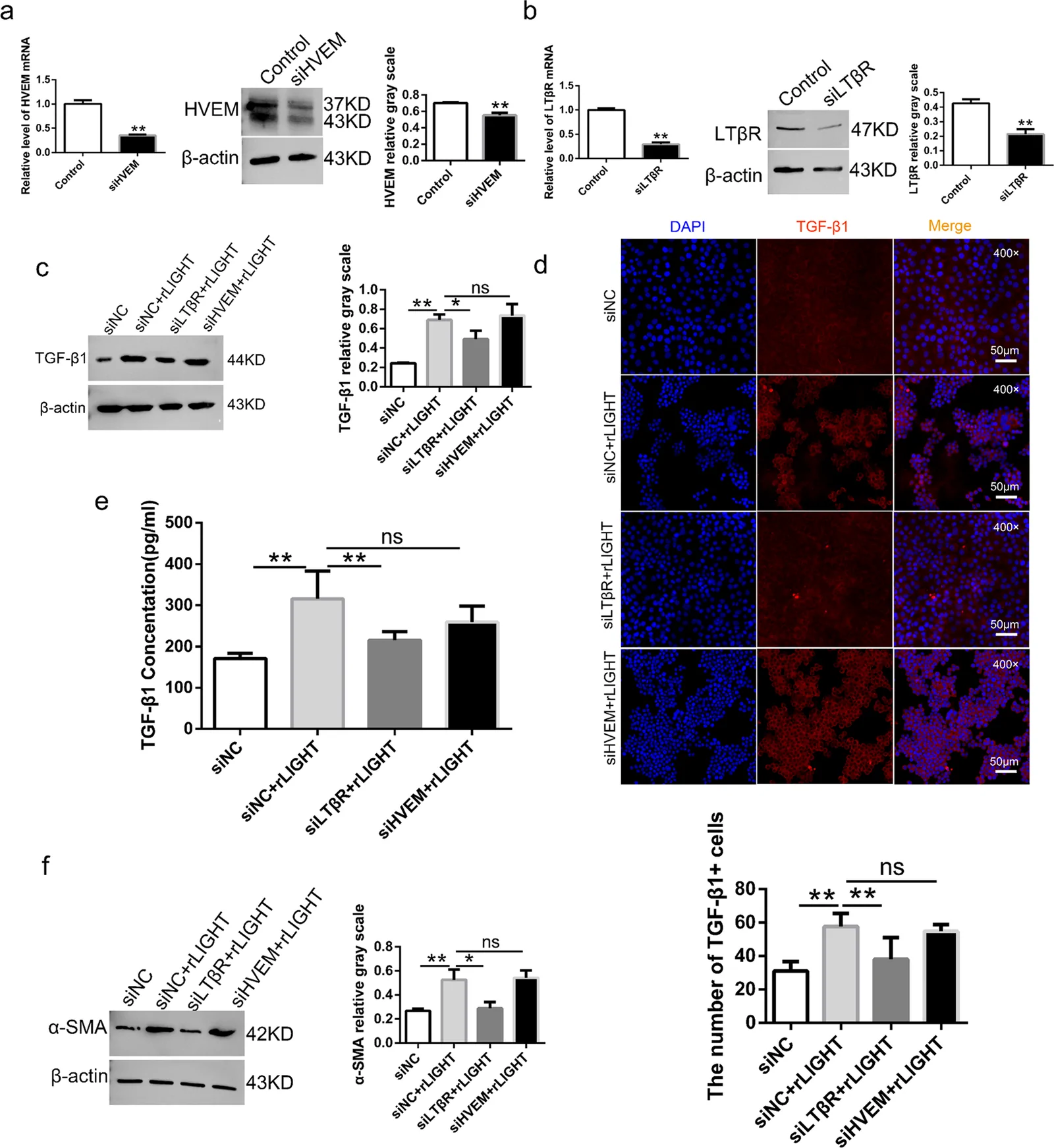
LIGHT uses LTβR but not HVEM to drive liver fibrosis. **a** The levels of HVEM after transfection with siHVEM. **b** The levels of LTβR after transfection with siLTβR. **c** Expression of TGF-β1 in RAW264.7 cells in the different groups. **d** Immunofluorescence staining of TGF-β1 and the number of TGF-β1-positive cells in RAW264.7 cells in the different groups. Magnification: ×400. **e** Concentration of TGF-β1 in JS1 cell culture medium, as determined by ELISA. **f** Expression of αSMA in JS1 cells in the different groups. siNC (transfection with control nonsense siRNA), siHVEM (transfection with HVEM siRNA), siLTβR (transfection with LTβR siRNA). Scale bar, 50 µm. The mRNA expression in RAW264.7 cells was examined by qPCR and normalized to GAPDH expression. The relative grayscale value of the protein band was measured with ImageJ software and normalized to β-actin. The data (means ± SEM) were obtained from triplicate experiments (unpaired two-sample Student’s *t*-test and one-way ANOVA with Tukey’s multiple comparison test, *P < 0.05 and **P < 0.01).

LIGHT has two receptors in the TNFR superfamily (LTβR/TNFRSF3 and HVEM/TNFRSF14^19^), both of which are expressed by macrophages^15^. To determine whether LIGHT drove liver fibrosis through one or both of its receptors, siRNAs against murine LTβR and HVEM and nonsense siRNA were transfected into RAW264.7 cells for 48 h, and then qPCR was used to measure LTβR and HVEM mRNA expression. As a result, the levels of both LTβR and HVEM were significantly reduced (*P* < 0.01) (Fig. 5a, b). These results indicated a successful knockdown of the target genes. Next, rLIGHT was used to stimulate RAW264.7 cells transfected with LTβR and HVEM siRNA. Compared with those in the SiNC group, the levels of TGF-β1 and αSMA were significantly upregulated in the SiNC + LIGHT groups (*P* < 0.01). Compared with that in the SiNC + LIGHT group, the expression of TGF-β1 and/or αSMA was significantly downregulated in rLIGHT-treated RAW264.7 cells transfected with LTβR siRNA, and there was no significant difference in the HVEM siRNA group (Fig. 5c–f). Taken together, these results suggest a mechanism by which LIGHT promotes fibrosis through direct binding to LTβR but not HVEM on hepatic macrophages to increase TGF-β1 expression.

### LIGHT directly induces liver fibrosis via TGF-β1

**Fig. 6:**
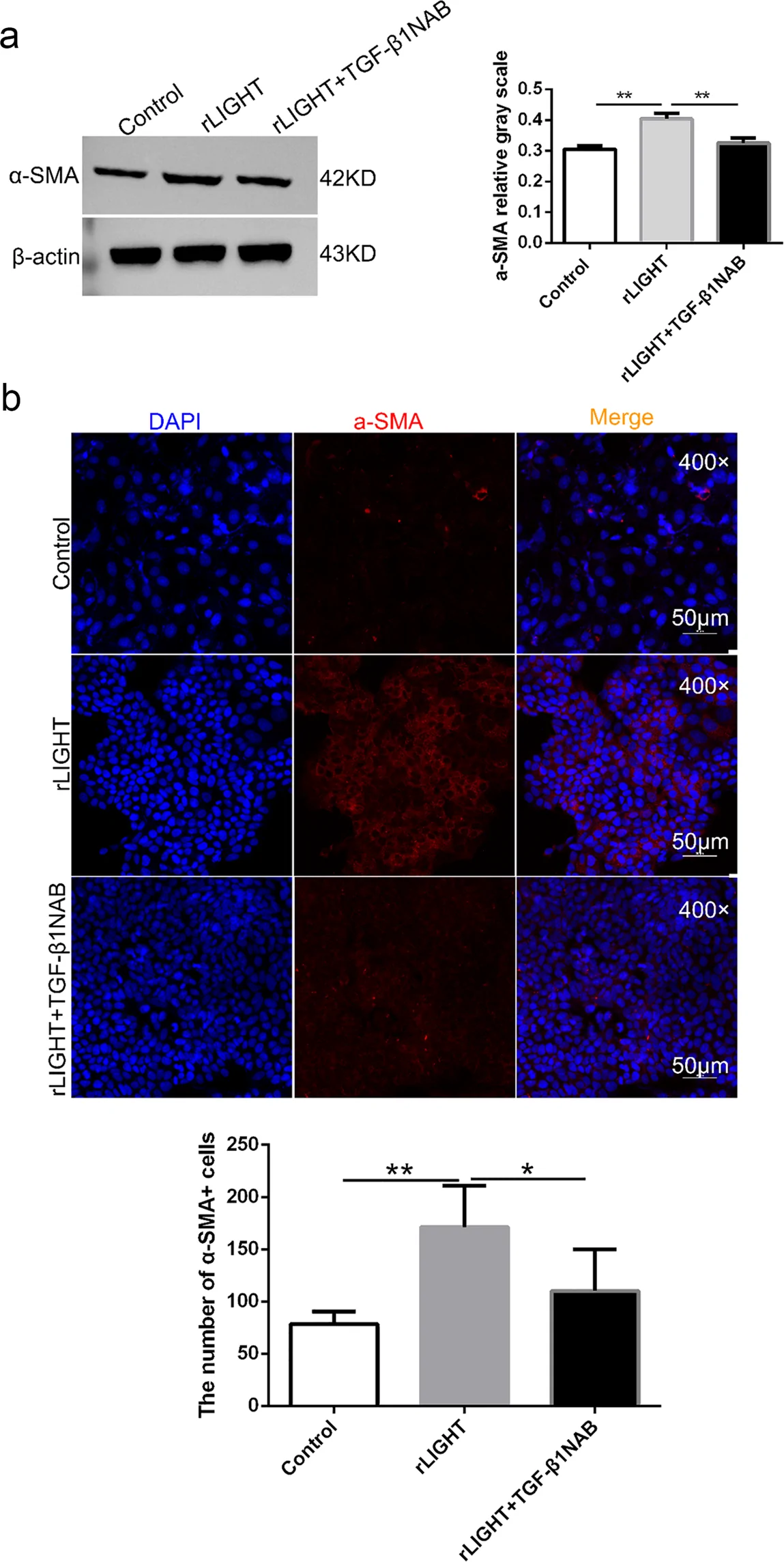
LIGHT directly induces liver fibrosis via TGF-β1. **a** The level of αSMA in JS1 cells after treatment with an anti-TGF-β1-neutralizing antibody. **b** Immunofluorescence staining of αSMA in JS1 cells after treatment with an anti-TGF-β1-neutralizing antibody. Magnification: ×400. Control (coculture assay of RAW264.7 and JS1 cells); rLIGHT group: (coculture assay of RAW264.7 and JS1 cells with rLIGHT stimulation); rLIGHT+TGF-β1NAB: (coculture assay of RAW264.7 and JS1 cells treated with rLIGHT and an anti-TGF-β1-neutralizing antibody); scale bar, 50 µm. The relative grayscale value of the protein band was measured with ImageJ software and normalized to β-actin. The data (means ± SEM) were obtained from triplicate experiments (one-way ANOVA with Tukey’s multiple comparison test, *P < 0.05 and **P < 0.01).

Murine hepatic macrophages are capable of producing TGF-β1, which participates in the development of liver fibrosis^26,27^. Therefore, we determined whether LIGHT induced liver fibrosis in vitro by a TGF-β1-dependent mechanism. We used an anti-TGF-β1-neutralizing antibody in the RAW264.7/JS1 cell coculture system to neutralize the effects of TGF-β1 after treatment with rLIGHT. Surprisingly, both western blotting and immunofluorescence showed decreased expression of αSMA in JS1 cells (Fig. 6a, b). Thus, LIGHT induced liver fibrosis through TGF-β1-dependent signaling.

### LIGHT promotes fibrotic factors in vivo

**Fig. 7:**
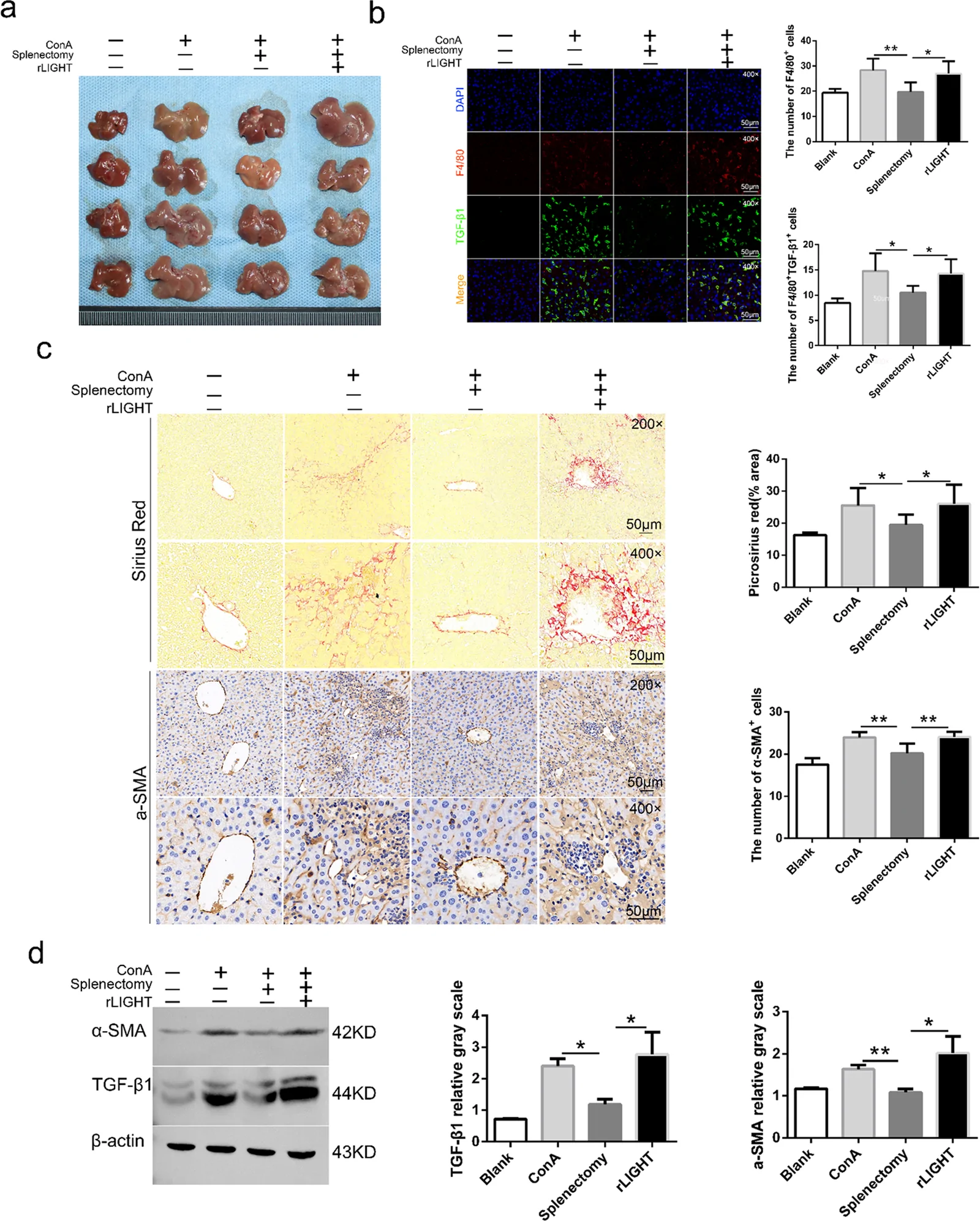
Role of LIGHT in liver fibrosis in vitro. **a** Gross images of liver tissues. **b** F4/80+ and F4/80+/TGF-β1 + macrophages were analyzed by immunofluorescence staining. Ratios were calculated (magnification ×400). **c** Sirius red staining and immunohistochemical staining of αSMA in the liver tissues in each group (magnification ×400 and ×200). **d** The levels of TGF-β1 and αSMA in the various groups. Normal mice (Blank), ConA-treated mice (ConA); ConA + splenectomy-treated mice (splenectomy); ConA + splenectomy + rLIGHT-treated mice (rLIGHT). In the animal studies, *n* = 6/group. In all panels, scale bars = 50 µm. The relative grayscale value of the protein band was measured with ImageJ software and normalized to β-actin. The data (means ± SEM) were obtained from triplicate experiments (one-way ANOVA with Tukey’s multiple comparison test, *P < 0.05, **P < 0.01).

Splenectomy improved liver fibrosis and induced a significant decline in the serum LIGHT level; thus, we assessed whether LIGHT had direct roles in exacerbating liver fibrosis after splenectomy in vivo. First, rLIGHT was administered through the angular vein to mice with liver fibrosis induced by ConA. Compared with those in the Blank group, hepatic volume was increased, and liver surfaces were gray and granular after ConA and rLIGHT treatment (Fig. 7a). Compared with those in the splenectomy group, F4/80+ and F4/80+/TGF-β1+ macrophages were significantly increased after rLIGHT treatment (*P* < 0.05) (Fig. 7b), and worsened liver fibrosis was indicated by Sirius red staining and immunohistochemical staining with a large area of fibrosis after rLIGHT treatment (Fig. 7c). Furthermore, the expression of TGF-β1 and αSMA was increased in the rLIGHT group (*P* < 0.01) (Fig. 7d). Therefore, rLIGHT increased liver fibrosis in mice that underwent splenectomy.

### Liver fibrosis is suppressed in LIGHT-deficient animals

**Fig. 8:**
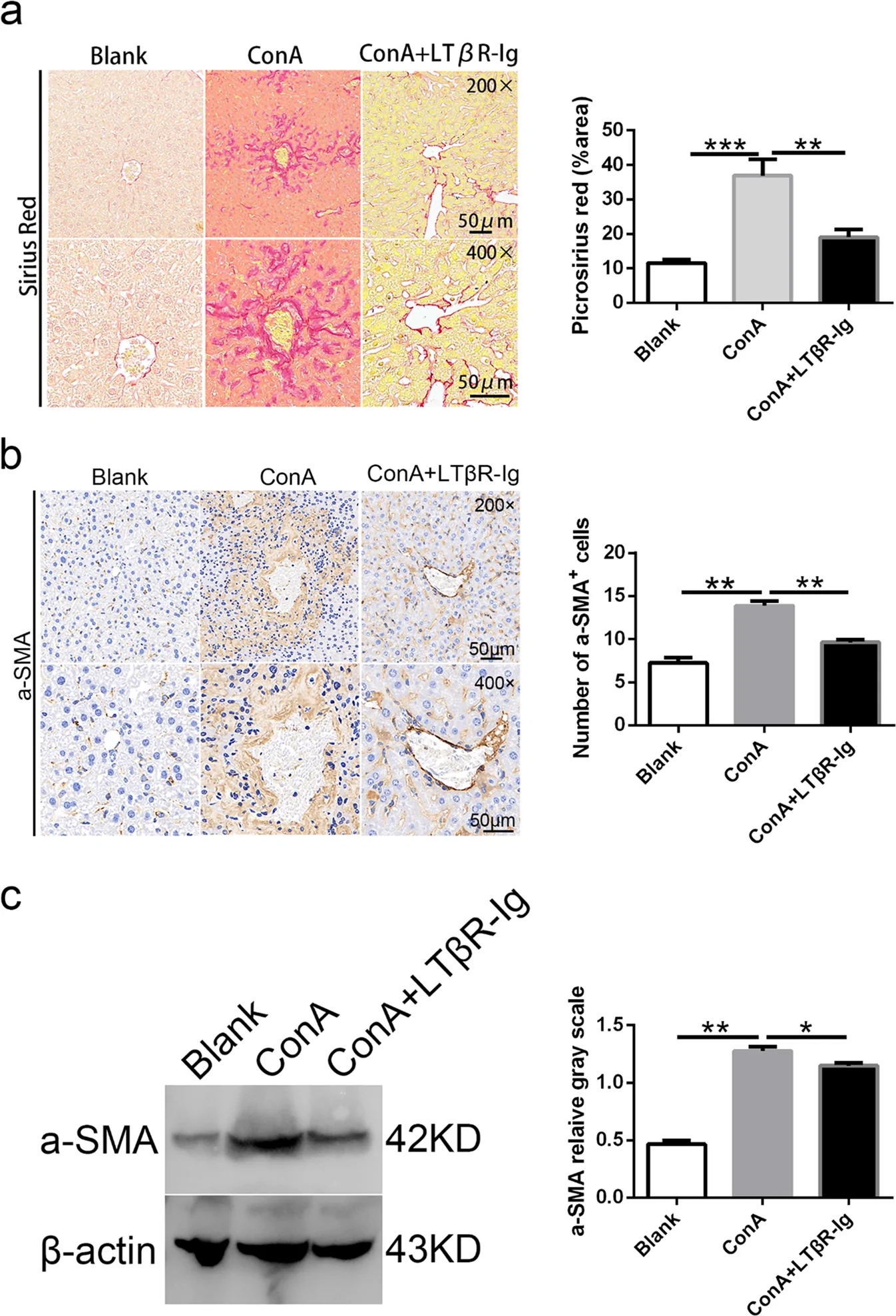
Histological analysis and the level of α-SMA in liver tissues. **a** Sirius red staining in liver tissues. **b** Immunohistochemical staining of α-SMA in liver tissues. **c** The levels of α-SMA in liver fibrosis. Normal mice (Blank), ConA-treated mice (ConA), ConA- and LTβR-Ig-treated mice (ConA+LTβR-Ig). In the animal studies, *n* = 3/group. In all panels, scale bars = 50 µm. The relative grayscale value of the protein band was measured with ImageJ software and normalized to β-actin. The data (means ± SEM) were obtained from triplicate experiments (one-way ANOVA with Tukey’s multiple comparison test, *P < 0.05, **P < 0.01, ***P < 0.001).

To better understand the effects of LIGHT on liver fibrosis, we used LTβR-Ig to block LIGHT in vivo and performed immunohistochemical staining, Sirius red staining, and western blotting to measure the level of αSMA and fibrosis in liver tissue samples. The results showed significantly decreased αSMA levels and fibrosis in the ConA+LTβR-Ig group (Fig. 8a–c).

## Discussion

Several reports have emphasized the protective effects of splenectomy on liver fibrosis^2,8,28–30^. However, the mechanism remains largely unknown. Here, we examined changes in profibrotic cytokines in the serum of cirrhosis patients before and after splenectomy. As profibrotic cytokines, LIGHT^15^, TGF-β1^29^, IL-6^31^, and TNF-α^32^ have major roles in fibrogenesis. We determined that LIGHT, TGF-β1, IL-6, and TNF-α were all significantly increased in the serum of cirrhotic patients, whereas splenectomy only induced significant decreases in LIGHT and TGF-β1. Therefore, splenectomy may be more closely associated with LIGHT and TGF-β1. LIGHT is mainly expressed by activated T cells^33^. However, as the membrane-bound form of LIGHT may be rapidly cleaved in the serum, the cellular and tissue sources of LIGHT can be difficult to identify^34^. Unfortunately, we have no data on the source of LIGHT in serum. Other studies have shown that LIGHT is increased in the serum of patients with various fibrotic and inflammatory diseases^34–37^. Additionally, LIGHT promotes the deposition of collagen in the context of fibrosis by controlling major profibrotic factors such as TGF-β1 and is considered to be a potential target to halt the development of fibrosis^15,19,34^. To verify the clinical associations among splenectomy, LIGHT, and TGFβ-1, we used a ConA-induced mouse liver fibrosis model. Similar to a previous study of the same model^30^, we also observed significant increases in serum ALT and AST levels and hepatic levels of TGF-β1 and αSMA and a significant decrease in PLT in the blood. More importantly, the serum level of LIGHT gradually increased during ConA treatment but decreased following splenectomy. By analyzing cirrhosis patients and an animal model of liver fibrosis, we found that splenectomy induced changes in LIGHT, TGF-β1, and liver fibrosis.

Many studies emphasize the relationship between splenectomy, macrophages, and liver fibrosis. For example, Wang et al.^30^ found that splenectomy suppressed the progression of liver fibrosis by affecting the polarization of MDSDs and M2 macrophages. Li et al.^12^ reported that splenectomy improved liver fibrosis by inhibiting the establishment of the M1-dominant phenotype in hepatic macrophages. Yada et al.^2^ found that splenectomy promoted Ly-6C^lo^ macrophage/monocyte accumulation in the fibrotic liver tissue of mice to improve liver fibrosis. Moreover, several effects related to LIGHT and macrophages have been reported in the development of tissue fibrosis. LIGHT induces the accumulation of macrophages and upregulates the level of TGF-β1 secreted by macrophages in asthmatic airway remodeling^16^. Other studies have shown that LIGHT acts on macrophages in a membrane-bound form to participate in fibrotic activity^17^. We suspected that splenectomy may also control the expression of LIGHT to affect the secretion of TGF-β1 by macrophages in the context of liver fibrosis. We found that rLIGHT affected the hepatic macrophage cell line RAW264.7, promoting the secretion of TGF-β1 and upregulating the level of P-JNK in these cells. In the splenocyte and RAW264.7 cell coculture system, blocking LIGHT in splenocytes significantly downregulated the expression of TGF-β1. JNK signaling is considered a major modulator of liver fibrosis^38^. Kou et al.^39^ reported that LIGHT inhibited JNK signal activation in beige biogenesis while activating NF-κB signaling. As an important modulator of the transcription factor AP-1, JNK activation might have a critical role in LIGHT-mediated cellular responses^40^. In our experiment, JNK phosphorylation was increased concomitant with the upregulated expression of TGF-β1. The JNK inhibitor strongly reduced TGF-β1 expression in RAW264.7 cells and the culture supernatant of JS1 cells. Correspondingly, the level of αSMA in JS1 cells was also decreased. These results showed that the decrease in fibrosis after splenectomy was directly related to reduced levels of LIGHT in vitro. LIGHT promoted the expression of TGF-β1 by activating JNK signaling, and the inhibition of JNK abrogated the effects of rLIGHT.

LIGHT has two receptors, HVEM and LTβR, both of which are expressed by macrophages. More importantly, these macrophages often reside close to subepithelial regions where smooth muscle hyperplasia and fibrosis often occur^15^. LIGHT is a major directive protein in the development of pulmonary tissue fibrosis and skin fibrotic activity, and inhibiting the binding of LIGHT to its receptors LTβR and HVEM improve fibrosis^15,18,19^. To determine whether LIGHT bound to HVEM and/or LTβR in the context of liver fibrosis, we transfected RAW264.7 cells with siRNAs. In a coculture system of HVEM or LTβR, siRNA-transfected RAW264.7 and JS1 cells treated with rLIGHT, we found that silencing LTβR downregulated the expression of TGF-β1 in RAW264.7 cells and αSMA in JS1 cells, while no significant difference was observed by silencing HVEM. Therefore, LIGHT bound to LTβR but not HVEM in RAW264.7 cells to induce the expression of TGF-β1 and αSMA and subsequently induce liver fibrosis. Furthermore, TGF-β1 neutralization in vitro revealed that TGF-β1 was important for the fibrotic effect of rLIGHT, which was consistent with studies that showed a central role of TGF-β in directly promoting fibrosis^41,42^. These findings expand our understanding of the complex regulatory networks among LIGHT, LTβR, JNK, and TGF-β1.

Although there was a promotional effect on liver fibrosis in vitro, the role of LIGHT in vivo remains unclear. Herro et al. found that rLIGHT injection upregulated the major profibrotic factors TGF-β, IL-13, and TSLP and had major roles in pulmonary and skin fibrosis. LIGHT-deficient mice also exhibit defective fibrotic activity in the skin^16,18,19^. Because splenectomy improved liver fibrosis induced by ConA, we examined whether splenectomy had the same effect after the injection of rLIGHT. If rLIGHT had a direct profibrotic role in liver fibrosis, splenectomy would not improve liver fibrosis after the injection of rLIGHT. The results showed that the injection of rLIGHT into mice exacerbated fibrosis in liver tissue and abolished the effect of splenectomy. Furthermore, blockade of LIGHT in animal models of liver fibrosis without splenectomy significantly improved liver fibrosis. These results illustrated the direct profibrotic role of LIGHT in liver fibrosis.

Our results suggest that LIGHT promotes liver fibrosis by binding to LTβR to activate the phosphorylation of JNK. As a result, the secretion of TGF-β1 by macrophages increases, resulting in liver fibrosis. Furthermore, serum LIGHT levels were reduced after splenectomy and indirectly improved fibrosis in liver tissue.

## Supplementary information

Supplemental Materials
