# Author Correction: Splenectomy improves liver fibrosis via tumor necrosis factor superfamily 14 (LIGHT) through the JNK/TGF-β1 signaling pathway

**DOI:** 10.1038/s12276-026-01833-w

**Published:** 2026-09-25

**Authors:** Qing-shan Liang, Jian-Gang Xie, ChaoPing Yu, ZhuSheng Feng, JingChang Ma, Yuan Zhang, Dong Wang, JianGuo Lu, Ran Zhuang, Jikai Yin

**Affiliations:** 1 Department of General Surgery, The Second Affiliated Hospital of Air Force Military Medical University, 710038 Xi’an, Shaanxi China; 2 Department of Emergency, The First Affiliated Hospital of Air Force Military Medical University, 710032 Xi’an, Shaanxi China; 3 Department of Immunology, School of Basic Medical Sciences, Air Force Military Medical University, 710032 Xi’an, Shaanxi China; 4 Transplant Immunology Laboratory, School of Basic Medical Sciences, Air Force Military Medical University, 710032 Xi’an, Shaanxi China; 5 Institute of Medical Research, Northwest Polytechnic University, 710072 Xi’an, Shaanxi China

Correction to: *Experimental & Molecular Medicine*

https://doi.org/10.1038/s12276-021-00574-2, published online 03 March 2021

After online publication of this article, an error was noticed in the ConA+Splenectomy group of Fig.2g. The images originally assigned to the Blank group and the ConA+Splenectomy group were all incorrectly extracted from figures of the Blank group, owing to the similarly fluorescence expression levels between these two groups. The figure should have appeared as shown below.

The authors apologize for any inconvenience caused.

**Figure:**
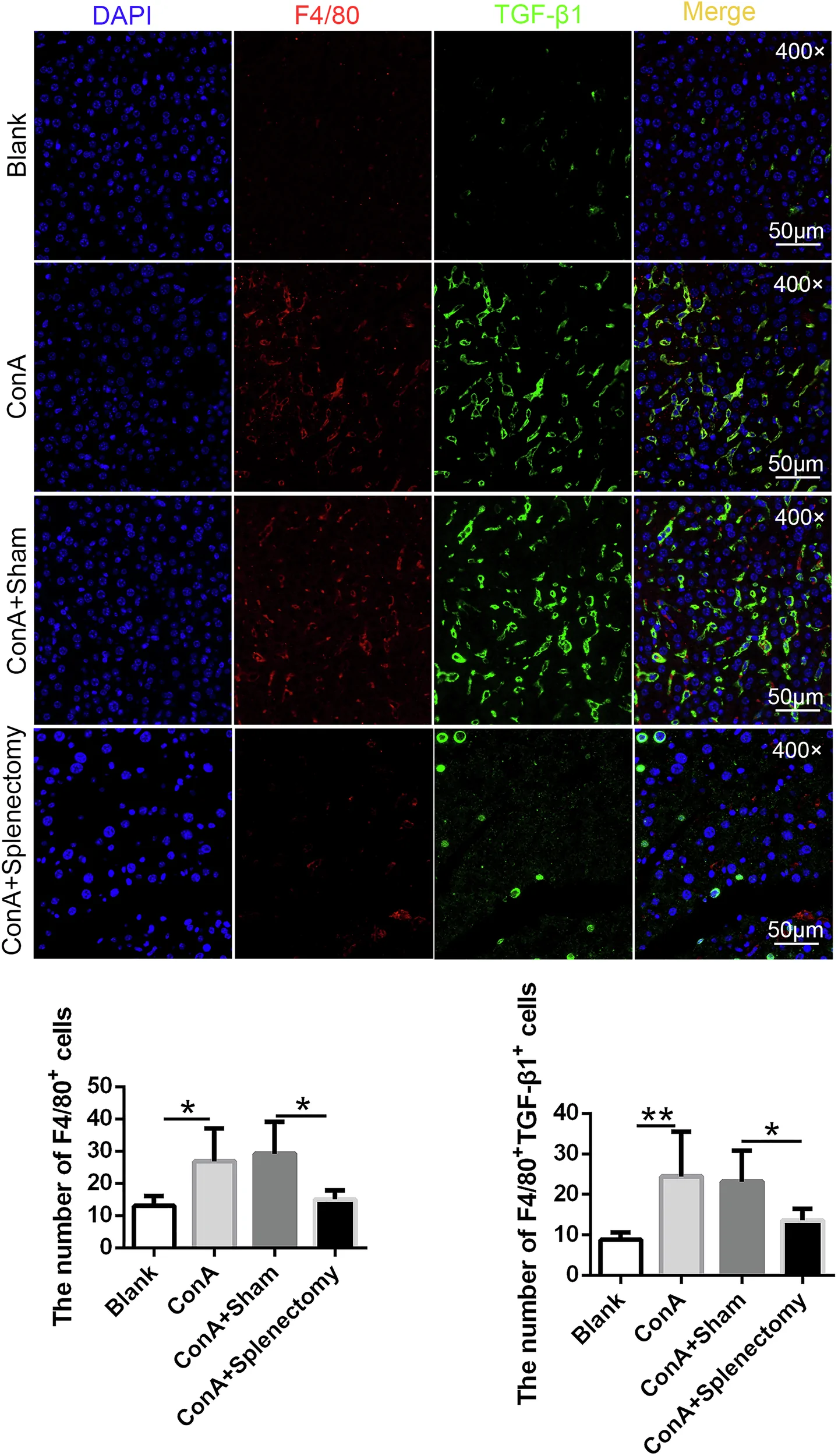

The original article has been corrected.

