# Supplementary material for: Splenectomy improves liver fibrosis via tumor necrosis factor superfamily 14 (LIGHT) through the JNK/TGF-β1 signaling pathway
Source: Exp Mol Med. 2021 Mar 3;53(3):393–406. doi: 10.1038/s12276-021-00574-2 (PMC8080781; doi:10.1038/s12276-021-00574-2)
Supplement: Supplementary file 1 — Supplemental Materials [file 12276_2021_574_MOESM1_ESM.docx]

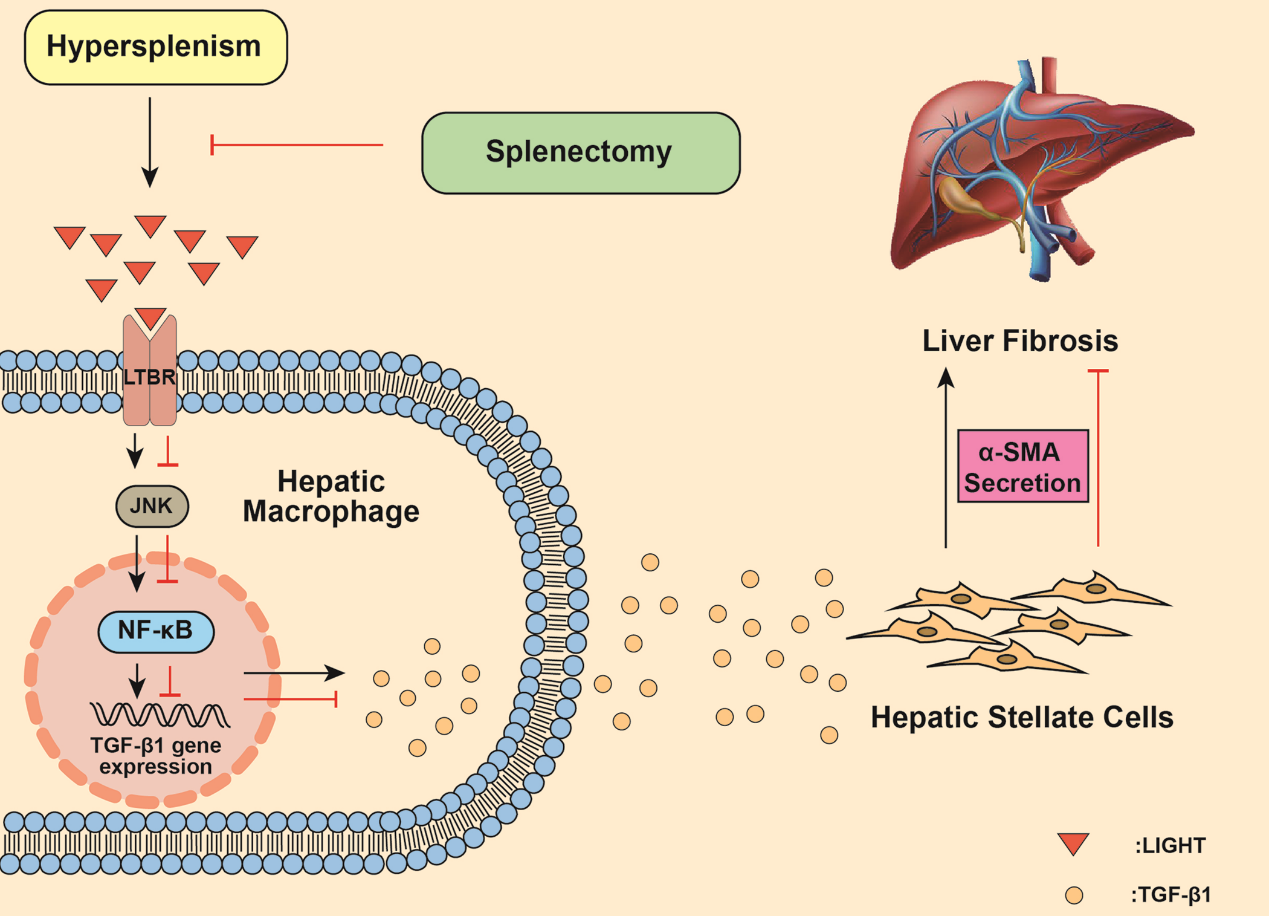


**Supplementary figure 1. Graphical abstract**

**Supplementary table 1.** **Clinical data for healthy volunteers,** **cirrhotic patients Pre and Post splenectomy**

| Clinical data | Healthy  （n=23） | Pre-splenectomy  （n=23） | Post-splenectomy（n=23） |
| --- | --- | --- | --- |
| Sex(M/F) | 9/14 | 10/13 | 10/13 |
| Ages(years) | 43.22±1.714 | 40.22±2.203 | 40.22±2.203 |
| Degree of esophageal varices(n,%) |  |  |  |
| Mild/Moderate/Severe | -- | 3(13.1)/5(21.7)/15(65.2) | -- |
| Esophageal varices bleeding(Y/N,%） | -- | 7(30.4%)/16(69.6%) | N |
| Child-Pagh分级（n，%） |  |  |  |
| ClassA/B/C | -- | 17(73.9)/6(26.1)/0(0) | 19 (82.6%)/4(17.4%) |
| platelet (PLT) | 197.2±14.60 | 52.78±4.307 | 274.5±21.99 |
| serum bilirubin(μmol/L) | 9.963±0.9111 | 29.38±3.449 | 20.49±1.442 |
| serum albumin(g/L) | 42.83±0.7936 | 40.14±1.103 | 47.79±9.904 |
| prothrombin time(s) | 11.38±0.2533 | 14.33±1.091 | 13.29±0.3506 |
| Ascites(Y/N,n,%) | -- | N | N |
| Alanineaminotransferase  （IU/L） | 23.90±3.396 | 35.91±2.998 | 30.13±2.717 |

**Supplementary table 2****. Oligo sequences for RT-PCR**

| Genes | Forward | Reverse |
| --- | --- | --- |
| GAPDH | 5′-TCAACGGCACAGTCAAGG-3′ | 5′-ACTCCACGACATACTCAGC-3′ |
| HVEM | 5′-TGTCCCCCACAGACATATACC-3′ | 5′-CTCACAGAAGTAGCCTGGGAT-3′ |
| LTβR | 5′-GCCCCTGTGACATTGTGCT-3′ | 5′-GGAAGCTTCGGGATTTATGG-3′ |
| TGF-β1 | 5′-GGAAGCTTCGGGATTTATGG-3′ | 5′-GGAAGCTTCGGGATTTATGG-3′ |
